# Mating systems and recombination landscape strongly shape genetic diversity and selection in wheat relatives

**DOI:** 10.1093/evlett/qrae039

**Published:** 2024-08-12

**Authors:** Concetta Burgarella, Marie-Fleur Brémaud, Gesa Von Hirschheydt, Veronique Viader, Morgane Ardisson, Sylvain Santoni, Vincent Ranwez, Miguel de Navascués, Jacques David, Sylvain Glémin

**Affiliations:** CNRS, Univ. Montpellier, ISEM – UMR 5554, Montpellier, France; AGAP Institut, Univ Montpellier, CIRAD, INRAE, Institut Agro, Montpellier, France; Department of Organismal Biology, Evolutionary Biology Center, Uppsala University, Uppsala, Sweden; AGAP Institut, Univ Montpellier, CIRAD, INRAE, Institut Agro, Montpellier, France; Swiss Federal Research Institute WSL, Birmensdorf, Switzerland; AGAP Institut, Univ Montpellier, CIRAD, INRAE, Institut Agro, Montpellier, France; AGAP Institut, Univ Montpellier, CIRAD, INRAE, Institut Agro, Montpellier, France; AGAP Institut, Univ Montpellier, CIRAD, INRAE, Institut Agro, Montpellier, France; AGAP Institut, Univ Montpellier, CIRAD, INRAE, Institut Agro, Montpellier, France; UMR CBGP, Univ Montpellier, CIRAD, INRAE, Institut Agro, Montpellier, France; AGAP Institut, Univ Montpellier, CIRAD, INRAE, Institut Agro, Montpellier, France; CNRS, Univ. Rennes, ECOBIO – UMR 6553, Rennes, France; Department of Ecology and Evolution, Evolutionary Biology Center, Uppsala University, Uppsala, Sweden

**Keywords:** self-fertilization, polymorphism, linked selection, fitness effect of mutations, selfing syndrome

## Abstract

How and why genetic diversity varies among species is a long-standing question in evolutionary biology. Life history traits have been shown to explain a large part of observed diversity. Among them, mating systems have one of the strongest impacts on genetic diversity, with selfing species usually exhibiting much lower diversity than outcrossing relatives. Theory predicts that a high rate of selfing amplifies selection at linked sites, reducing genetic diversity genome-wide, but frequent bottlenecks and rapid population turn-over could also explain low genetic diversity in selfers. However, how linked selection varies with mating systems and whether it is sufficient to explain the observed difference between selfers and outcrossers has never been tested. Here, we used the *Aegilops*/*Triticum* grass species, a group characterized by contrasted mating systems (from obligate outcrossing to high selfing) and marked recombination rate variation across the genome, to quantify the effects of mating system and linked selection on patterns of neutral and selected polymorphism. By analyzing phenotypic and transcriptomic data of 13 species, we show that selfing strongly affects genetic diversity and the efficacy of selection by amplifying the intensity of linked selection genome-wide. In particular, signatures of adaptation were only found in the highly recombining regions in outcrossing species. These results bear implications for the evolution of mating systems and, more generally, for our understanding of the fundamental drivers of genetic diversity.

## Introduction

How and why genetic diversity varies among species is a central and long-standing question in evolutionary biology, dating back to the 1960s (Ellegren & Galtier, 2016). For neutral variation, patterns of genetic diversity depend on the balance between mutation and genetic drift, characterized by the effective size of a population, *N*_e_, and also on the efficacy of selection for functional regions of the genome. Recently, thanks to the availability of population genomic data in many nonmodel species, several studies have explored the ecological correlates of diversity levels, usually measured as nucleotide polymorphism, 𝜋. These surveys have shown that life history traits (LHTs), especially life-span and reproductive mode, can explain a large part of the observed variation in genetic diversity among species (Chen et al., 2017; Mackintosh et al., 2019; Muyle et al., 2021; Romiguier et al., 2014). LHTs may reflect long-term effective population size, which depends on current population size and past fluctuations across generations (e.g., Mackintosh et al., 2019; Romiguier et al., 2014). Effective population size can also depend on selection at linked sites, i.e., the hitch-hiking effect of the fixation of beneficial or the removal of deleterious mutations on linked neutral variation (Cutter & Payseur, 2013), which also affects long-term *N*_*e*_ and seems rather pervasive across genomes (Buffalo, 2021; Chen et al., 2020; Corbett-Detig et al., 2015; Mackintosh et al., 2019).

Among LHTs, mating systems deeply affect the genetic and ecological functioning of a species and are predicted to strongly impact both demographic outcomes and the response to selection. Thanks to the ability to produce seeds under limited mate availability, the capacity of autonomous selfing provides reproductive assurance and can be an ecologically successful strategy, allowing colonizing new habitats and increasing species range (Grossenbacher et al., 2015), which should be associated with a large census population size. However, being able to reproduce alone implies much higher demographic stochasticity due to recurrent bottlenecks and colonization-extinction dynamics, which strongly reduces genetic diversity, not only at the population scale but also at the whole species scale (Ingvarsson, 2002; Pannell & Charlesworth, 1999). Moreover, the dynamics of range expansions, which can be associated with the evolution of selfing, can also unintuitively lead to the loss of diversity, especially on the expansion front (Excoffier et al., 2009). So, despite possible large species range and census population size, the specific ecology of selfing species may lead to a reduction in *N*_e_. In addition to these demographic effects, selfing also has direct genetic effects that can reduce *N*_e_. Nonindependent gamete sampling during mating automatically increases genetic drift and reduces genetic mixing, which generates genome-wide genetic linkage disequilibrium, enhancing the effect of linked selection (Agrawal & Hartfield, 2016; Hartfield & Bataillon, 2020; Roze, 2016).

So far, striking differences in genetic diversity have already been observed between outcrossing and selfing relatives (e.g., Burgarella et al., 2015; Hazzouri et al., 2013; Slotte et al., 2013; Teterina et al., 2023) and the underlying causes (linked selection, demographic instability) have been often discussed and studied from a theoretical point of view (Barrett et al., 2014; Charlesworth et al., 1993), but, to our knowledge, attempts at a direct quantification with empirical data are recent and only partial (see the comparison between two outcrossing and selfing *Caenorhabditis* species in Teterina et al., 2023). Yet, how the intensity of linked selection varies with the mating system and whether it can be sufficient to explain the observed difference between outcrossing and selfing species remains to be quantified. Beyond a genome-wide reduction in polymorphism and selection efficacy with increasing selfing rates, the theory also predicts that genomic patterns across chromosomes should vary with the interaction between recombination and selfing rates. We expect a clear positive relationship between genetic diversity and recombination in outcrossers but an increasingly flatter relationship in species with increasing selfing rates. We also expect that deleterious mutations should accumulate mainly in lowly recombining regions, whereas adaptation should be prevalent in highly recombining regions in outcrossing species, in contrast to selfing species where signatures of high deleterious load and low adaptation should be more evenly distributed along the genome.

**Figure 1.**
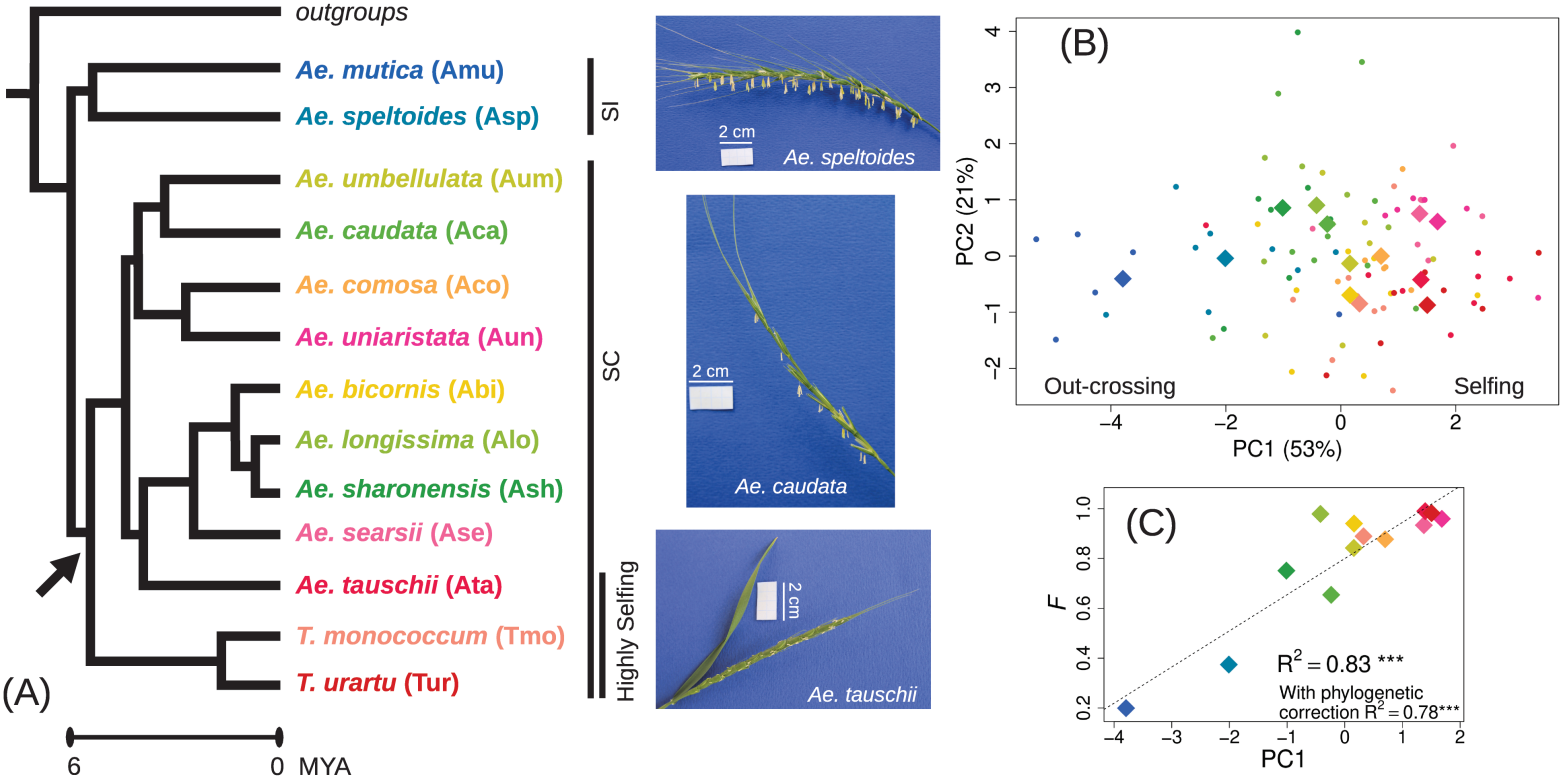
Mating system diversity in the genus *Aegilops*/*Triticum*. (A) Phylogenetic relationships among the 13 diploid species of the genus, following Glémin et al. (2019b). Colors have been assigned to represent a gradient of self-fertilization: *Ae. speltoides* and *Ae. mutica* have previously been described as self-incompatible (SI), *Ae. tauschii* and *Triticum* sp. are known to be prevalently self-fertilizing species (Highly Selfing), while the remaining species are self-compatible (SC). The arrow indicates when self-compatibility may have appeared if we assume that it appeared once according to a parsimony approach. Pictures illustrate the phenotypes associated with different mating systems: SI *Ae. speltoides*, highly selfing *Ae. tauschii* and SC *Ae. caudata*. (B) Principal Component Analysis of morphological measures of reproductive organs. PC1 resumes the morphological selfing syndrome. On one side, there are the SI species, which show bigger anther and stigmas, higher male investment, lower spikelet compactness, and lower autonomous seed set. On the other side, there are predominately selfing species with opposite states of traits. (C) Positive correlation between PC1 of reproductive traits and the inbreeding coefficient *F* estimated on the genomic data shows that species with stronger morphological selfing syndrome also have higher *F* values.

Here, we tested these hypotheses by comparing species with a large range of mating systems occurring within a single genus, which allows strong genetic contrast among otherwise similar species, an advocated sampling design (Cutter & Payseur, 2013; Leffler et al., 2012). We used the *Aegilops*/*Triticum* grass species as a study system. This group of Mediterranean and Western/Central Asian grasses belongs to the Triticeae tribe (Poaceae) and includes wheat and its wild relatives. The *Aegilops*/*Triticum* genus forms a monophyletic group with 13 diploid and about 17 polyploid species that likely diversified around 4–7 million years ago (Glémin et al., 2019b; Huang et al., 2002; Marcussen et al., 2014). All species are characterized by similar life history traits (wind-pollinated, annual, herbaceous species) and ecology (open landscapes, warm-temperate climate) but present a large diversity of mating systems, spanning from obligate outcrossing to highly selfing species (van Slageren, 1994; Kilian et al., 2011) (Figure 1). Triticeae genomes are large, with markedly U-shaped recombination patterns along chromosomes conserved across species: most recombination is located in the distal parts whereas no or very low recombination occurs in their central part (Brazier & Glémin, 2022). Marked differences in both mating systems among species and recombination rate within genomes make the group an ideal model to unravel the role of selection on species genetic diversity.

Through a comparative population genomic approach, we assessed the expectation that genetic diversity and selection efficacy decrease with a higher selfing rate and with a lower recombination rate within each species by controlling for species range that varies among species (Supplementary Figure S1). We then explicitly tested whether the effect of linked selection was stronger in selfing species as predicted by population genetics theory. We found that selfing strongly affects genetic diversity and the efficacy of selection by amplifying the intensity of linked selection genome-wide, while species range plays a minor role. We also showed that genomic patterns remarkably matched the gradient of mating systems across species, while models and empirical evidence so far suggested that only extreme mating systems left clear signatures in the genomes (Agrawal & Hartfield, 2016; Roze, 2016). These results have multiple implications for both the evolution of mating systems and our understanding of the fundamental drivers of genetic diversity.

## Methods

### Plant material

We analyzed molecular and morphological data of the 13 extant wild diploid species of the *Aegilops*/*Triticum* genus (Triticeae tribe, Kilian et al., 2011) and three outgroup species (*Taeniatherum caput-medusae*, *Hordeum spontaneum*, and *Secale strictum*) for a total of 98 accessions. We targeted 7–20 individuals from each of five focal species, including the two self-incompatible (SI) outcrossing *Ae. speltoides* and *Ae. mutica* and the three predominantly selfing *Ae. tauschii*, *T. urartu*, and *T. monococcum*. We included 2–4 accessions from each of the other *Aegilops*/*Triticum* species and 1–3 from each of the outgroups. The accessions were obtained from several international seed banks and donor researchers. The list of accessions per species with their passport information is provided in Supplementary Table S1.

### Morphological data

To finely characterize the selfing syndrome of each species, we measured several morphofunctional traits describing the reproductive organs and function (Escobar et al., 2010 and references therein; Friedman & Harder, 2005). Around 3–4 grains per accession were sown in January 2014 in a greenhouse. After emergence, the seedlings were submitted to 4 °C for 6 weeks to ensure vernalization requirements. Only one seedling per accession was kept after vernalization. The first two spikes of each plant were closed in paper bags to prevent cross-fertilization. We collected the two bagged spikes, one open mature spike, mature anthers, stigmas, and ovaries for measurements. Anthers, stigmas, and ovaries were preserved in the Carnoy fixative solution. For each of 5–7 accessions per *Aegilops*/*Triticum* species and 1–3 accessions per outgroup species, we measured a mature spike (length, spikelet number, grain number), three spikelets per spike (spikelet length) and three flowers from one spikelet (length of palea and lemma). All flowers of the three spikelets were classified as fertile (if the presence of grain was observed), female (only stigma observed), male (anthers observed), or sterile. For each accession, we measured six anthers (length and width) and three stigmas and ovaries (length of each organ). For anthers, stigma, and ovaries, each organ was measured five times, and the mean value of these replicates was used for further analysis. Measures were manually recorded on millimeter paper or taken photographs with the software analySIS (Soft Imaging System GmbH 2002; see Supplementary Figure S12 for an example).

Missing data (31%) on the directly observed measures were imputed with the missMDA package (Josse & Husson, 2016) under the R environment (R Core Team, 2018). Parameters were set by default, and the optimal number of components retained for imputation was estimated with the cross-validation method (ncp = 3). Raw and imputed measures are provided in Supplementary Tables S2 and S3, respectively, and the list of measured traits is provided in Supplementary Table S4.

Additional variables were calculated on imputed measures as follows. The mean values of anther and stigma dimensions were calculated per accession and standardized by dividing by the flower length. Following Escobar et al. (2010), the autonomous seed set was estimated as self-fertilised_seed_number/(self-fertilized_spikelet_number*number_fertile_flower/spikelet), corresponding to the number of seeds per fertile flower. Spikelet compactness was calculated as the ratio (mean flower length* number_fertile_flower)/mean spikelet length. Male investment was calculated as the ratio of the mean anther length and the mean ovary length. We used these additional variables to summarize the selfing syndrome with a synthetic measure corresponding to the first axis of a Principal Component Analysis (PCA) (Supplementary Tables S3 and S4). The PCA was performed with the ade4 package (Dray & Dufour, 2007) under the R environment.

### Species range

We expect that species with bigger census sizes also harbor higher genetic diversity, a relationship that could mask or interact with the effect of the mating system. To control for this potential effect, we used species range as a proxy for census size since, to our knowledge, there are no direct estimates of census size for wild *Aegilops*/*Triticum* species. To estimate species range, we retrieved occurrence data from the Global Biodiversity Information Facility (http://www.gbif.org) for each species. We manually cleaned the data set to remove single occurrences outside the species range, which can be due either to identification errors or recent introductions. Cleaned data were mapped on the world map (focusing on western Eurasia and North Africa) on which we applied a grid with a cell size of one decimal degree square (~10,000 km^2^). We estimated species range as the number of cells occupied by a species time 10,000 km^2^ (Supplementary Figure S1 and Supplementary Table S5).

### Sequencing

We added 48 new sequences to the dataset used for the phylogenomic analysis of Glémin et al. (2019b) and Clément et al. (2017), for a total of 98 sequences for 13 *Aegilops*/*Triticum* species (*n* = 2–21) and 3 outgroup species (*n* = 1–3).

We performed full transcriptome sequencing following the procedure described in Sarah et al. (2017) and Glémin et al. (2019b). Briefly, RNAs were extracted and prepared separately for leaves and inflorescence tissues and mixed subsequently in 20% and 80% proportions, respectively. RNA was extracted using a Spectrum Plant Total RNA kit (Sigma-Aldrich, USA) with a DNAse treatment. RNA concentration was measured with two methods: a NanoDrop ND-1000 Spectrophotometer and the Quant-iT™ RiboGreen (Invitrogen, USA) protocol. RNA quality was assessed on the RNA 6000 Pico chip on a Bioanalyzer 2100 (Agilent Technologies, USA). Following the Illumina TruSeq mRNA protocol, we kept samples with an RNA Integrity Number value greater than eight. Libraries were prepared with a modified protocol of the TruSeq Stranded mRNA Library Prep Kit (Illumina, USA) to obtain library fragments of 250–300 bp. Modification details and amplification conditions are available in Glémin et al. (2019b). After verifying and quantifying each indexed cDNA library using a DNA 100 Chip on a Bioanalyzer 2100, pooled libraries were made of 12 equally represented genotypes. Each final pooled library was quantified by qPCR with the KAPA Library Quantification Kit (KAPA Biosystems, USA) and sequenced using the Illumina paired-end protocol on a HiSeq3000 sequencer by the Get-PlaGe core facility (GenoToul platform, INRA Toulouse, France http://www.genotoul.fr).

### Transcriptome assembly, mapping, and genotype calling

Reads cleaning and assembly were performed with the pipeline described in Sarah et al. (2017). Adapters were removed with cutadapt (Martin, 2011). Reads were trimmed at the end, removing sequences with low-quality scores (parameter −q 20), and we retained only reads with a minimum length of 35 bp and a mean quality higher than 30. Orphan reads were then discarded using a homemade script. Retained reads were assembled with ABySS (Simpson et al., 2009), using the paired-end option with a kmer value of 60, followed by one step of Cap3 (Huang & Madan, 1999) run with the default parameters, 40 bases of overlap, and 90% percentage of identity. To predict the CDS embedded in our contigs, we used the *prot4est* program (Wasmuth & Blaxter, 2004). We provided three gene datasets: the output of a *Rapsearch* (Ye et al., 2011) similarity analysis, *Oryza* matrix model for de novo-based predictions, and the codon usage bias observed in *T. monococcum*. We run Rapsearch to identify protein sequences similar to our contigs in either the plant species of Uniprot swissprot (http://www.uniprot.org) or in the Monocotyledon species of greenphyl (http://www.greenphyl.org/cgi-bin/index.cgi). For the individual used as a mapping reference within each species (see below), we discarded predicted CDS with less than 250bp.

For each species, mapping was done with bwa (Li & Durbin, 2009) option –mem (instead of –aln) more adapted for reads of 100 bp. Reads were mapped on the sequences of the individual with the highest coverage or with the highest number of annotated contigs. The list of samples used as reference sequences and the total number of contigs per reference is given in Supplementary Table S5.

For each individual, diploid genotypes were called with reads2snps v. 2.0.64 (Gayral et al., 2013; Tsagkogeorga et al., 2012) (available at https://kimura.univ-montp2.fr/PopPhyl/index.php?section=tools). This tool is specifically designed to analyze transcriptome data for population genomics of nonmodel species. The method first calculates the posterior probability of each possible genotype in the maximum-likelihood framework after estimating the sequencing error rate. Genotypes supported with a probability higher than a given threshold (here 0.95) are retained; otherwise, missing data are called. We required a minimum coverage of 10× per position and per individual to call a genotype. SNPs are then filtered for possible hidden paralogs (duplicated genes) using a likelihood ratio test based on explicit modeling of paralogy (“paraclean” option embedded in the reads2snps software; Gayral et al., 2013). First, genotype and SNPs were called assuming panmixia (heterozygote deficiency *F* = 0), and *F* was estimated on the retained SNPs. As we have species-wide samples, *F* is equivalent to a *F*_IT_ and mainly corresponds to *F*_IS_ for selfing species and to *F*_ST_ for outcrossing ones. As the assumed expected heterozygosity can affect genotype calling and paralog filtering, reads2snps were run a second time for each species using the *F* estimated after the first step. For the outgroup species with sample size *n* = 1 (*T. caput-medusae*), we kept the initial genotype calling and filtering procedure.

Open-reading frames were predicted using the program ORF_extractor.pl (available at https://kimura.univ-montp2.fr/PopPhyl/index.php?section=tools). Gene length and number of SNPs in the final data set per species are provided in Supplementary Table S5.

Orthologous pairs of open-reading frames, hereafter called genes, from the five focal and one outgroup species (*T. caput-medusae*) were identified using reciprocal best hits on BLASTn results, a hit being considered as valid when *e*-value was below e-50. Outgroup sequences were added to within-focal species alignments using MACSE v. 1.2 (Ranwez et al., 2011), a program dedicated to the alignment of coding sequences and the detection of frameshifts. Genes were only retained if no frameshift was identified by MACSE and if the predicted ORF in the focal species was longer than 100 codons.

### Chromosome patterns and recombination map

We wanted to analyze polymorphism patterns across chromosomes and as a function of recombination rates. Unfortunately, there is neither a reference genome nor a genetic map available for every species. Among the high-quality recombination maps available (see Brazier & Glémin, 2022), we first used the recombination map of *Hordeum vulgare* as a reference for all species. The synteny is well conserved at the scale of Triticeae (Mayer et al., 2011; but see Parisod & Badaeva, 2020) and, as *H. vulgare* is an outgroup, there should not be a specific bias for one species or another. For comparison, we also used the three constitutive genomes AA, BB, and DD of the hexaploid wheat, *Triticum aestivum*, which correspond to the three main lineages in the *Aegilops*/*Triticum* phylogeny (Glémin et al., 2019b and Figure 1). These genomes are closer to the focal species, but the phylogenetic distance depends on the genome and the species.

To build the recombination maps, we built genetic versus physical distance maps (Marey’s maps). For the barley genome, we used the genetic SNP markers from Comadran et al. (2012), which were initially mapped on version 082214v1 of the barley genome. We thus used the coordinate correspondence between this first version and the new reference genome assembly (Hv_IBSC_PGSB_v21) to locate the SNP markers on this reference genome. After visual inspection of aberrant markers, we kept a total of 3,590 markers (on average ~513 markers per chromosome). Recombination rates were computed with the *MareyMap* R package (Rezvoy et al., 2007) by fitting a loess function with a second-degree polynomial on sliding windows containing 20% of the markers of a chromosome. This led to a rather smooth recombination map, which is sufficient for our purpose of capturing large-scale patterns and reducing noise. For the bread wheat genome, we used the Marey maps built in Brazier & Glémin (2022). Genetic distances were then interpolated between markers using the fitted function so that a genetic distance could be attributed to each annotated gene of the *Hordeum* genome. For each gene, we computed the local recombination rate by taking the local derivative of the fitted loess function as implemented in MareyMap (recombination maps are provided in Supplementary Figure S13).

For each assembled transcript of each focal species, we searched for its orthologous sequence in the *H. vulgare* genome (H) using reciprocal best blast and retaining pairs when the *e-*value was below e-50. Then, the two sequences were aligned with MACSE v.1.2, and the synonymous divergence, *D*_S_, was computed using *codeml* from the PAML software (Yang, 2007). The *D*_S_ distribution was clearly bimodal for all species, and gene pairs showing too high divergence (*D*_S_ > 0.35) were discarded as they likely corresponded to paralogues. For each transcript in each focal species, we attributed the same genetic distance and local recombination rate as its ortholog in *H. vulgare*. We applied the same procedure for the three *T. aestivum* subgenomes (A, B, and D) separately, except that we did not filter on *D*_S_ that cannot be used as a homogeneous criterium for all species as the distance depends on the subgenome and the focal species, contrary to *Hordeum*, which as the same expected distance with all focal species.

For each focal species, to assess the similarity among genomes, we counted how many genes had an orthologue on the same chromosome of the four reference genomes (H, A, B, and D) and whether it corresponded to the same category of recombination (low: below the median, or high: above the median).

### Sequence polymorphism analysis

Polymorphism and divergence statistics were calculated with dNdSpNpS v.3. (available at https://kimura.univ-montp2.fr/PopPhyl/index.php?section=tools) that rely on the Bio++ libraries (Guéguen et al., 2013). Further filters were applied to the data sets. Positions at which a genotype could be called in less than five individuals for species with sample size *n* ≥ 5 and in less than *n*/2 for species with *n* < 5 were discarded. Genes with less than ten codons were discarded. For each gene, the following statistics were calculated: per-site synonymous (𝜋_S_) and nonsynonymous (𝜋_N_) mean pairwise nucleotide diversity, heterozygote deficiency (*F*), number of synonymous (S_S_) and nonsynonymous (S_N_) segregating sites, number of synonymous (*D*_S_) and nonsynonymous (*D*_N_) fixed differences between focal and outgroup species. These statistics were computed from complete, biallelic sites only, i.e., sites showing no missing data after alignment cleaning and no more than two distinct states. For each species, statistics were averaged across genes, weighting by the number of complete sites per gene, thus giving equal weight to every SNP. For 𝜋_N_/𝜋_S_ and *D*_N_/*D*_S_, we first computed the averages of 𝜋_N_, 𝜋_S_, *D*_N_, and *D*_S_ and subsequently the ratios of averages. Confidence intervals were obtained by 10,000 bootstraps over genes. For the focal selfing species *Ae. tauschii*, *T. monococcum*, and *T. urartu*, all statistics were calculated on *n*/2 alleles by randomly drawing one haploid sequence per gene and individual (Supplementary Table S5).

### Fit of a linked selection model

To go further, we fitted a linked selection model, following Corbett-Detig et al. (2015) and Elyashiv et al. (2016) but including the effect of partial selfing. To simplify the model, and because we did not have information about substitutions across the genome, we only considered background selection. The genome was split into genomic windows. For each region where $\pi_{S}\left(i\right)$ has been estimated on *n*_*i*_ positions, we assumed that *n*_*i*_ × $\pi_{S}\left(i\right)$ followed a binomial distribution with parameter *n*_*i*_ and *p*_*i*_ given by:


$$p_{i}=\pi_{max}e^{-\sum_{j}^{}u_{j}\frac{s}{{\left(r_{ij}+s\right)}^{2}}}$$ (1)

where *s* is the mean selection coefficient against deleterious mutations, *r*_*ij*_ represents the probability of recombination between the focal region *i* and any other region of the genome, *j* containing *L*_*j*_ coding positions so that *u*_*j*_* = u L*_*j*_, and where *u* is the rate of deleterious mutations. To improve the fit to the data, we considered a distribution of fitness effects (DFE) of mutations. We used a simple discrete distribution with three categories characterized by their mutation rates and selection coefficients: *u*_*1*_, *u*_*2*_, *u*_*3*_, and *s*_*1*_, *s*_*2*_, *s*_*3*_. *Equation 1* can thus be generalized as:


$$p_{i}=\pi_{max}e^{-\sum_{j}^{}L_{j}\left(u1\frac{s1}{{\left(r_{ij}+s1\right)}^{2}}+u2\frac{s2}{{\left(r_{ij}+s2\right)}^{2}}+u3\frac{s3}{{\left(r_{ij}+s3\right)}^{2}}\right)}$$ (2)

Note that the *s* values correspond, in fact, to (*h + F—hF*)*s* where *h* is the dominance coefficient. However, because our aim is not to estimate and compare deleterious mutation parameters, we did not fit *h* and *s* separately. Recombination probability, *r*_*ij*_, is obtained from the genetic distance, *d*_*ij*_, using Haldane’s mapping function: *r*_*ij*_ = (1—exp(−2*d*_*ij*_))/2. To take partial selfing into account, we rescaled *r*_*ij*_ by *r*_*ij*_(1—*F*) (see Nordborg, 2000). This rescaling is correct only for low-recombination rates and a more accurate expression of background selection was obtained by Roze (2016). However, the simple rescaling provides a good approximation and is much simpler to handle than the full expression (Roze, 2016). Note that we took the sum on all genomic regions on the same chromosome of the focal region but also on other chromosomes (so with *r*_*ij*_ = ½). This is especially important under partial selfing as selection on one chromosome can also affect the other chromosomes. Under outcrossing, it boils down to the effect of the additive variance in fitness that reduces effective population size (Roze, 2016).

We had seven parameters to estimate: *u*_*1*_, *u*_*2*_, *u*_*3*_, *s*_*1*_, *s*_*2*_, *s*_*3*_, and $\pi_{max}$. Because estimates of *F* were not very precise, we ran the model by letting *F* free and being estimated jointly with the other parameters (so eight parameters in total). The log-likelihood function was optimized with the *optim* function in *R* using the “L-BFGS-B” method and the constraint: *u*_*1*_ > *u*_*2*_ > *u*_*3*_.

Recombination is very heterogeneous along chromosomes (U-shaped), and large central regions are strongly linked. Instead of splitting the genome into regions of equal physical size (in Mb), we split it into regions of equal genetic size (in cM) based on the Marey map interpolation. Genomic regions on the telomeric parts of the chromosomes were thus shorter than centromeric regions, where recombination is very low. We chose a window size of 1 cM, and we discarded regions with less than 300 bp to avoid too noisy data. To obtain confidence intervals on parameter estimations, we bootstrapped data 100 times and rerun the model.

### Linear models and phylogenetic correction

We looked at the relation of polymorphism (𝜋_S_, 𝜋_N_/𝜋_S_, and $\pi_{max}$) and *F* with mating system (PC1 of reproductive morphology) and species geographical range using simple unweighted linear regressions of the form *y* ~ *x* (*F* ~ PC1, log(𝜋_S_) ~ PC1, 𝜋_N_/𝜋_S_ ~ PC1, log(𝜋_S_) ~ range, 𝜋_N_/𝜋_S_ ~ range, 𝜋_N_/𝜋_S_ ~ *F*). To evaluate the joint effects of mating system and species range on polymorphism (𝜋_S_ and 𝜋_N_/𝜋_S_), we performed multiple linear regressions in the form log(𝜋_S_) ~ mating_system + species range and 𝜋_N_*/*𝜋_S_ ~ mating_system + species range. We then represented the residuals, after removing the effect of the mating system, as a function of species range. All linear models were run with the function *lm* under the R environment.

We also applied a correction to take into account the phylogenetic relationships among species using the ultrametric tree retrieved from Glémin et al. (2019b). For this, we computed the phylogenetically independent contrasts using the method of Felsenstein (1985) with the package *ape* version 5.6-2 (Paradis & Schliep, 2019). The function *pic* was applied to the *y* and *×* vectors with default parameters, and then the *lm* analysis was repeated with the contrast values obtained instead of the raw values.

### Estimation of the DFE of mutations

In the two SI species, *Ae. mutica* and *Ae. speltoides*, and in two highly selfing species, *Ae. tauschii* and *T. urartu*, we estimated the DFE of mutations using the *PolyDFE* method (Tataru & Bataillon, 2019; Tataru et al., 2017). In brief, this method used the unfolded site frequency spectrum (uSFS) for both synonymous and nonsynonymous mutations to fit the distribution of fitness effect (DFE) of mutations modeled by the mix of a gamma distribution for deleterious mutations and an exponential distribution for beneficial mutations. Demography is taken into account by adding and fitting noise parameters that distort uSFS from the equilibrium expectation following Eyre-Walker et al. (2006). Because uSFS are sensitive to polarization errors, which can give spurious signatures of beneficial mutations, a probability of mis-polarization is also added and fitted in the model. This yields a set of four related models: with and without beneficial mutations and with and without polarization errors. Instead of choosing the best model to estimate parameters, we ran all four and used a model averaging procedure (as in Muyle et al., 2021): each parameter estimate was averaged using Akaike weights $e^{\frac{-1}{\Delta AIC_{i}}}$ with $\Delta AIC_{i}=AIC_{i}-AIC_{min}$ where $AIC_{min}$ is the AIC of the best model. Confidence intervals were obtained by bootstrapping SNPs 1,000 times.

For the four species, uSFS were polarized using *Taeniatherum caput-medusae* as an outgroup. We performed three analyses: on the whole set of SNPs or by splitting the dataset into two subsets: SNPs from genes in the high or low recombining regions. GC-biased gene conversion (gBGC), a recombination-associated process mimicking selection in favor of G and C nucleotides, is known to be active in grasses (Clément et al., 2017; Rodgers-Melnick et al., 2016) and could lead to spurious signatures of positive selection in highly recombining regions. To test for the possible effect of gBGC, we also rerun the analyses on three categories of SNPs: AT→GC, GC→AT, and G←→C + A←→T.

### Simulations

To understand how different selfing rates affect our ability to detect the effects of linked selection on polymorphism landscape and DFEs, we run forward-time individual-based evolutionary simulations in SLiM v.3.3 (Haller & Messer, 2019). We simulated a population of *N* = 10,000 individuals with five different selfing rates: 0, 0.5, 0.9, 0.99, and 1. We considered a genome of three Mb with a single chromosome composed of 1,000 genes of 1,000 bp separated by intergenic regions of 2,000 bp. Recombination decreased exponentially from 60 cM/Mb at the tips to 6 cM/Mb in the center of the chromosome, corresponding to a total genetic of 3.24 Morgan (so an average of three crossovers per chromosome per meiosis, which is in the range of one to three/four that is observed in plants, Brazier & Glémin, 2022). We assumed a mutation rate of 10^-6^, with ⅔ of mutations being neutral, corresponding to an expected genetic diversity of 4*Nu* = 0.027, of the order of magnitude of what we observed in the outcrossing species, and corresponding to an average *r*/*u* close to one. The other third of mutations were considered deleterious with a dominance level of *h* = 0.25 and deleterious effects in homozygotes drawn in a gamma distribution with mean = 0.01 and shape = 0.5. This corresponds to a genomic deleterious mutation rate of *U* = 0.33. After a burn-in period of 10*N* generations, we recorded the genome sequence of 15 individuals. We run ten replicates for each selfing rate.

We used the simulated data to assess the effect of selfing and linked selection on the estimation of the DFE. Importantly, polyDFE estimates the shape and the population-scaled mean of the DFE: *S* = 4*N*_e_(*h* + *F*—*hF*)*s*. However, *N*_e_ is not set nor fixed in the model, contrary to *h*, *s*, and *F*, but depends on the intensity of linked selection. We thus used the observed 𝜋_S_ divided by 4*u* to get the predicted *N*_e_, hence the predicted *S*.

## Results

### Mating system widely varies in *Aegilops*/*Triticum* genus

We analyzed phenotypic and transcriptomic diversity in 98 accessions from the 13 diploid *Aegilops*/*Triticum* species and three close outgroup species *Taenatherium caput-medusae*, *Hordeum vulgare,* and *Secale vavilovii* (Figure 1; Supplementary Table S1 and Supplementary Figure S1). Individuals were sampled over the whole geographic range to assess genetic diversity at the species scale. For some species the mating system was already well known, including the SI *Ae. speltoides* and *Ae. mutica* and the highly selfing *T. urartu*, *T. monococcum*, and *Ae. tauschii* (Dvořák et al., 1998; Escobar et al., 2010), but for others, it was poorly documented (Kilian et al., 2011). We thus characterized the mating system of each species by quantifying six floral and reproductive traits, including the size of female and male reproductive organs (anthers, stigmas), male investment, spikelet compactness, and the autonomous seed set (Escobar et al., 2010) (Supplementary Tables S2, S3, and S4). Building on previous work (Escobar et al., 2010), we considered these traits as indicative of the selfing syndrome, i.e., the specific changes in flower morphology and function that are expected to occur following the evolution of self-fertilization, especially for anemophilous species (Escobar et al., 2010; Sicard & Lenhard, 2011). The autonomous seed set provided a verification that SI species *Ae. mutica* and *Ae. speltoides* produced almost no seeds under imposed self-fertilization in the greenhouse (bagged spikes), while all the other species were able to produce seeds (Supplementary Figure S2). All the other traits were significantly negatively (anther and stigma size, male investment) or positively (spikelet compactness) correlated with the autonomous seeds set (Supplementary Figure S3), indicating that the selected traits are good indicators of the mating system of each species.

We summarized this reproductive morphofunctional diversity with a multivariate approach, PCA. The PCA first axis reflected the differences in mating systems within the *Aegilops*/*Triticum* genus (Figure 1B and Supplementary Figure S4). On one extreme of PC1 there were SI *Ae. mutica* and *Ae. speltoides*, which showed bigger anthers and stigmas, higher male/female investment, lower spikelet compactness, and lower (null) autonomous seed set. On the other extreme, there were the predominantly selfing *Ae. tauschii*, *T. urartu*, and *T. monococcum*, with opposite states of the traits (Figure 1A and B). The other *Aegilops* species showed intermediate values of the multi-trait statistic. This result still held when outgroups were included in the analysis (Supplementary Figure S5).

Species morphology measured by PC1 explained well the genome-wide estimate of inbreeding coefficient, *F*, calculated on the whole transcriptome dataset (*R*^2^ = 0.83, *p* = 1.68e-05, Figure 1C), confirming that our phenotypic data represented a good proxy of the mating system. Thus, in the following analyses, we used PC1 to describe the selfing syndrome, which avoids using genomic data for both characterizing the mating systems and their genomic consequences.

These findings are in agreement with previous knowledge (Dvořák et al., 1998; Escobar et al., 2010) and allowed us to characterize the mating system for the species that lacked outcrossing rate estimations. They also showed that the effects of selfing might be gradual, with species exhibiting mixed mating strategies described by intermediate values of *F* and of the phenoypic selfing syndrome. Mapping mating systems on the phylogeny suggested that self-incompatibility was likely ancestral and may have broken only once as all species are self-compatible (SC), except the two external ones (*Ae. mutica* and *Ae. speltoides*). However, several breakdowns of self-incompatibility cannot be excluded. For example, high selfing could have evolved four times independently in the branches leading to *Ae. tauschii*, *Ae. searsii*, *Ae. uniaristata* and *Triticum* species (Figure 1A).

### Polymorphism strongly correlates with mating systems

Genetic diversity was estimated for each species from whole transcriptome sequencing data. Sequences of 48 accessions were generated and de novo assembled in this study and were added to the datasets of Glémin et al. (2019b) and Clément et al. (2017) (Supplementary Table S1). Between 19,518 and 28,834 coding sequences were obtained per species. After filtering, genotype calling was performed on a number of contigs varying from 7,083 (*Ae. searsii*) to 21,706 (*T. caput-medusae*) (Supplementary Table S5).

**Figure 2.**
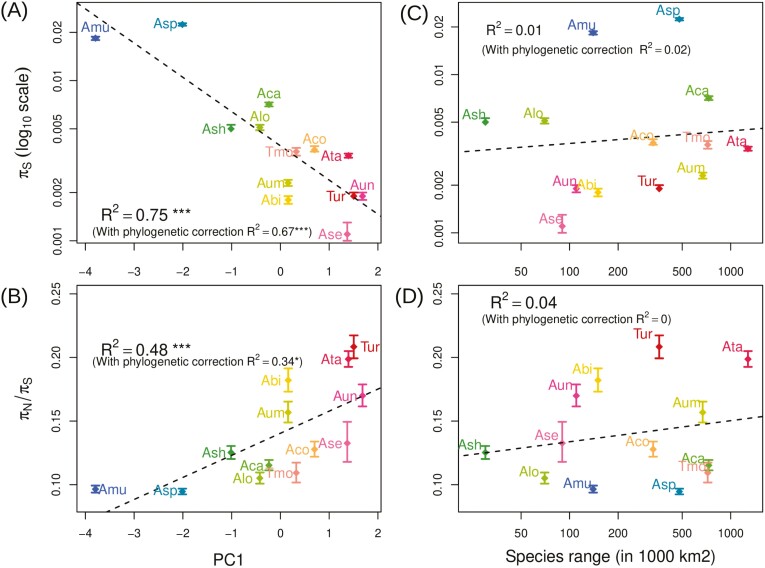
Mating systems traits and global polymorphism patterns. Neutral genetic diversity 𝜋_S_ is negatively correlated with the first axis of a Principal Component Analysis (PCA) of morphofunctional traits associated with reproduction (A), while the efficacy of selection estimated with 𝜋_N_/𝜋_S_ ratio is positively correlated with the same PC1 axis (B). Species geographical range estimated from GBIF occurrence data cannot explain the variation observed neither in 𝜋_S_ (C) nor 𝜋_N_/𝜋S (D).

Selfing is expected to reduce neutral genetic diversity (Ingvarsson, 2002; Jarne, 1995; Nordborg, 2000; Pollak, 1987; Schoen & Brown, 1991), here estimated as synonymous polymorphism, 𝜋_S_. Across species, 𝜋_S_ varied more than one order of magnitude, from 0.0011 for the SC *Ae. searsii* to 0.02 for the SI *Ae. speltoides*. According to expectations, genetic diversity decreases with increasing selfing rate. Neutral genetic diversity, 𝜋_S_ and PC1 were significantly correlated across species (*R*^2^ = 0.75, *p* = 0.00014), indicating a gradient in which stronger selfing syndrome corresponds to lower genome-wide neutral diversity (Figure 2A). The correlation was still significant after phylogenetic control (*R*^2^ = 0.67, *p* = 0.00111). Interestingly, this relationship is more or less log-linear (Figure 2A), with the main difference being observed between the two SI (*Ae. speltoides* and *Ae. mutica*) and the SC species (all the others).

Selfing is also expected to reduce the efficacy of selection, thus leading to a higher accumulation of segregating slightly deleterious mutations in selfing than in outcrossing species (Glémin, 2007). In agreement with this prediction, the efficacy of purifying selection estimated by the ratio of nonsynonymous to synonymous polymorphism 𝜋_N_/𝜋_S_ (Kimura, 1983) was lower for selfing species (max 𝜋_N_/𝜋_S_ value 0.21 for *T. urartu*) than for outcrossing ones (0.09 for *Ae. speltoides*). Similarly to neutral diversity, the efficacy of selection was also significantly explained by the selfing syndrome (*R*^2^ = 0.48, *p* = 0.0083; with phylogenetic control *R*^2^ = 0.35, *p* = 0.041; Figure 2B). We further verified that both polymorphism statistics, 𝜋_S_ and 𝜋_N_/𝜋_S_, also correlated with *F* estimates (Supplementary Figure S6).

We also tested whether species range, used as a proxy of census population size, also correlated with genetic diversity, with widespread species predicted to be more polymorphic than species with restricted geographic distribution. In contrast to the mating system, species range was not correlated with either 𝜋_S_ (Figure 2C) or 𝜋_N/_𝜋_S_ (Figure 2D). Such a correlation could be masked by the strong effect of selfing, which is expected to favor species range expansions. For example, *Ae. tauschii* is highly selfing and has by far the largest species range (Supplementary Figure S1). However, a linear model with the two effects showed that the mating system still significantly explained both 𝜋S (*p* = 0.00004) and 𝜋_N_/𝜋_S_ (*p* = 0.0157) whereas species range did not (Supplementary Figure S7). Yet, the effect of species range on 𝜋_S_ is barely significant (*p* = 0.063), so it is still possible that there is a weak effect that we could not detect with only 13 species. Overall, these results suggest that the mating system is the main driver of genetic diversity in *Aegilops*/*Triticum* species and overwhelms the potential effects of recent population history.

### The effect of linked selection depends on the mating system

We tested the hypothesis that selfing increases the effect of linked selection by comparing polymorphism patterns and recombination along chromosomes. Species-specific recombination maps were not available for most species, so we used the recombination map of the outgroup species *Hordeum vulgare*, which we compared to the recombination maps of the three diploid subgenomes of bread wheat (A, B, and D, corresponding to the wild parents *T. urartu*, *Ae. speltoides/mutica*, *Ae. tauschii*). For each focal species, we found that 99% or orthologs with *H. vulgare* mapped on the same chromosome as at least one of the three subgenomes of *T. aestivum*, and 96% to 97% as all three of them. Similarly, 97% to 99% of orthologs with *H. vulgare* belonged to the same recombination category as at least one of the three subgenomes and 78% to 79% as all three of them (Supplementary Table S6).

In what follows, we only show the results with *H. vulgare* since it provides the further advantage that the outgroup has (on average) the same phylogenetic distance to every *Aegilops*/*Triticum* species, ensuring an unbiased analysis that does not favor species closer to the reference. For comparison, some additional results using the *T. aestivum* subgenomes as reference are given in supplementary material (Supplementary Table S6 and Supplementary Figure S9).

**Figure 3.**
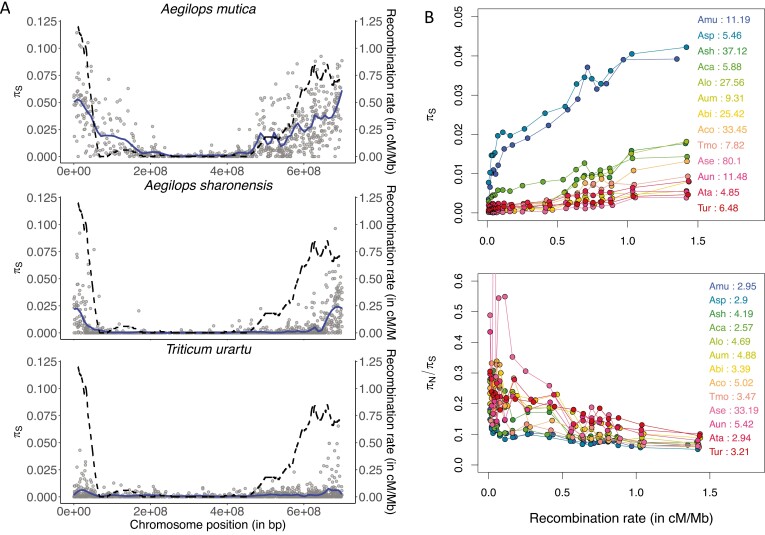
Recombination and patterns of genetic polymorphism across the genome. (A) Mean synonymous diversity (𝜋_S_) along chromosome 3 for three species with contrasted mating system: self-incompatible (*Ae. mutica*), mixed mating (*Ae. sharonensis*), and highly selfing (*T. urartu*); same scales for the three species. Each point corresponds to a contig mapped on the *Hordeum vulgare* genome. The solid line is the loess fitting function (degree = 2, span = 0.1). The dashed line indicates *H. vulgare* recombination map (in cM/Mb). (B) π_S_ and π_N_/π_S_ as a function of recombination rate. Contigs have been grouped in 20 quantiles of recombination. The value associated with each species corresponds to the ratio between the highest and the lowest value among the quantiles: from about 5 to 80 for π_S_ and from 2.5 to 33 for π_N_/π_S_. Curves correspond to loess fitting functions (degree = 2, span = 0.2).

In all species, synonymous polymorphism was strongly correlated with recombination and presented a U-shaped pattern along chromosomes, more or less mirroring the recombination pattern (see Figure 3A and Supplementary Figure S14). However, the higher the selfing rate, the flatter the relationship (Figure 3B), suggesting a strong effect of the mating system on the relationship between polymorphism and recombination. We verified that the positive relationship between diversity and recombination rate was not merely caused by the mutagenic effect of recombination by looking at the correlation between synonymous divergence (*D*_*S*_) with the outgroup (*H. vulgare*) and recombination rate (Kulathinal et al., 2008). We found that the magnitude of *D*_*S*_ variation (factor 1.5, Supplementary Figure S8) is much lower than the range of variation observed for polymorphism along the genome (factor 5 to 80, depending on the species, Figure 3B). If a mutagenic effect of recombination cannot be ruled out, it is clearly insufficient to explain the magnitude of the correlation between 𝜋_S_ and recombination. This mere observation suggested that linked selection could strongly reduce 𝜋_S_ by at least one or two orders of magnitude.

**Figure 4.**
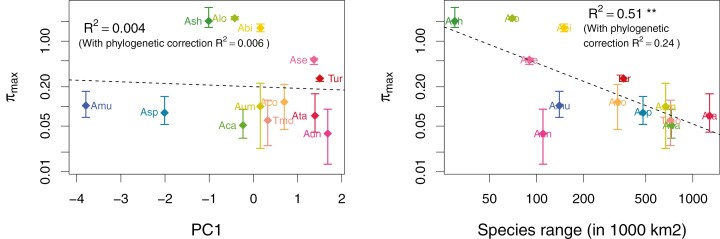
Relation of estimated maximum polymorphism in the absence of linked selection and mating system. 𝜋_max_ does not correlate with the first axis of the PCA of morphofunctional traits (left). 𝜋_max_ correlates negatively with species range (right), but the correlation is mainly driven by the species of the *Sitopsis* section and is no longer significant after phylogenetic correction.

To quantify more directly the effect of linked selection, we fitted a model similar to Corbett-Detig et al. (2015) and Elyashiv et al. (2016) that we adapted to partially selfing species but only considering background selection (see Supplementary Table S7 for full results). From the fit of the model, we obtained the maximum 𝜋_S_ that could be reached in the absence of linked selection, 𝜋_max_, which ranged between 0.028 to 5.82 (Figure 4). Note that, here 𝜋_max_ = 4*N*_e*_*max_*u*, so can be higher than one. We estimated that linked selection reduced 𝜋_S_ by 3.5 in *Ae. speltoides* and 5.6 in *Ae. mutica*, the two SI species. For other species, 𝜋_S_ was reduced by a few tens or even a few hundred (from 7 to 888), but without a clear relationship with the mating system. In contrast to 𝜋_S_, 𝜋_max_ did not correlate with PC1. Surprisingly, it correlated negatively with species range, but the correlation was mainly driven by the species of the *Sitopsis* section and was no longer significant after phylogenetic correction (Figure 4). It can be difficult to properly fit a realistic linked selection model for selfing species, and the results can be sensitive to the fact that we did not use the reference genome of each species. In particular, some 𝜋_max_ values were very high and could be overestimated, but fitting the model using the three subgenomes A, B, and D of *T. aestivum* gave similar results (Supplementary Figure S9). Overall, although they must be viewed with caution, the results strongly suggested that linked selection is a main driver of the effect of selfing on genetic diversity whereas species range has only a minor effect, and if any, not in the predicted direction. When recombination maps will be available in all species, it will be possible to reassess this result.

### Deleterious mutations accumulate, and adaptation is reduced under selfing and low recombination

Another central prediction of the effect of genetic linkage is that selection should be less efficient in genomic regions of low recombination, which can extend genome-wide in highly selfing species. In agreement with this expectation, the efficacy of purifying selection at the genome-wide level clearly decreased with the selfing rate (Figure 2B). All species also showed a negative relationship between recombination rates and the 𝜋_N_/𝜋_S_ ratio (Figure 3B), indicating that purifying selection was more efficient in highly recombining regions. More precisely, the 𝜋_N_/𝜋_S_ ratio sharply dropped with increasing recombination in outcrossing species but more and more smoothly with increasing selfing rate, which supports the prediction that reduced selection efficacy extended to larger genomic regions in selfing species.

**Figure 5.**
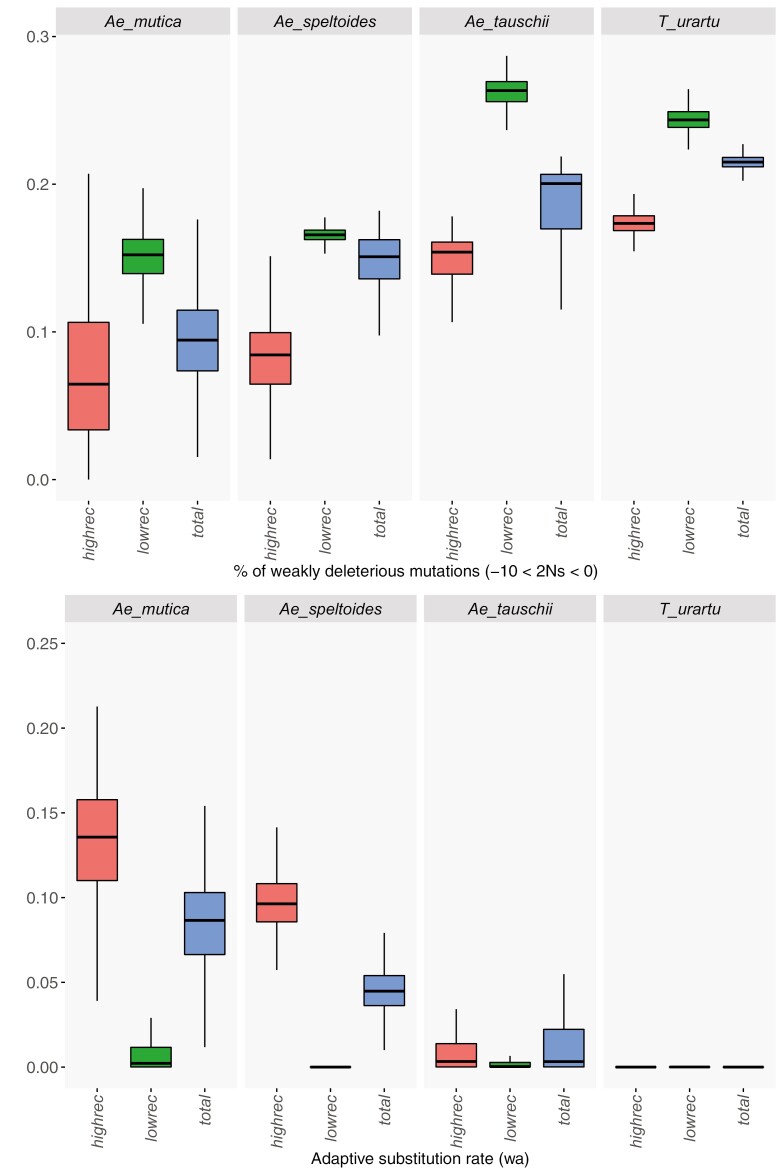
Mating systems, recombination, and the efficacy of selection. The distribution of the fitness effect of mutations was estimated in two self-incompatible species (*Ae. mutica* and *Ae. speltoides*) and in two highly selfing species (*A. tauschii* and *T. urartu*) at the whole genome scale (total) and for regions of low (lowrec) and high (highrec) recombination. (A) Proportion of weakly selected deleterious mutations (−10 < 2*N*_*e*_*s* < 0). (B) Rate of adaptive substitution, i.e., the expected *D*_N_/*D*_S_ ratio due to the flux of beneficial mutations.

The 𝜋_N_/𝜋_S_ ratio is a rather crude proxy for the efficacy of purifying selection and can be affected by several factors, such as nonequilibrium population dynamics, that can lead to the spurious signature of relaxed selection (Brandvain & Wright, 2016). To better characterize how selection efficacy varies with mating system and recombination, we estimated the full DFE of mutations, i.e., including both deleterious and beneficial mutations—using the *polyDFE* method (Tataru et al., 2017). This approach leverages information from unfolded synonymous and nonsynonymous site frequency spectra to infer the DFE of each species. It takes into account factors that can distort the SFS, such as nonequilibrium demography and linked selection, in addition to potential polarization errors. The method requires a sufficient number of chromosomes sampled (say >10), so we applied it only to the four species with the largest sample sizes, which correspond to the extreme mating systems used to calibrate the selfing syndrome: the two SI *Ae. speltoides* and *Ae. mutica* and two highly selfing *Ae. tauschii* and *T. urartu*. In agreement with the 𝜋_N_/𝜋_S_ ratio analysis, we found that the two selfers suffered from a higher load than the two outcrossers, with 22% to 25% of mutations not being efficiently selected against (−10 < *N*_e_*s* < 0) versus only 9% to 15% in the outcrossers (Figure 5). We also found a strong difference between regions of low and high recombination (higher vs. lower than the median) for all species. However, the difference was stronger in outcrossers (more than two-fold) than in selfers (30%–60% difference only) (Figure 5). Interestingly, purifying selection appeared as efficient in low-recombination regions of outcrossing genomes than in high-recombination regions of highly selfing genomes (Figure 5).

Another striking result is that we estimated an adaptive substitution rate not different from zero in the two selfers, but a quite high value in the two outcrossers and only in highly recombining regions (Figure 5). We verified that this signature of positive selection was not due to the spurious effect of GC-biased gene conversion (Supplementary Figure S10), which is known to happen in recombining regions of grass genomes (Muyle et al., 2011). Overall, selection appeared to be much less efficient, both on beneficial and against deleterious mutations, in low-recombination regions of outcrossing species and throughout the genome of highly selfing ones. When the proportion of weakly deleterious mutations is estimated with the DFE-alpha method (Keightley & Eyre-Walker, 2007) it was shown that selfing could overestimate it (Gilbert et al., 2022). Here, we used polyDFE, which was claimed to be less sensitive to linked selection effects (Tataru et al., 2017). In addition, the model assumes additive selection, whereas deleterious mutations are partially recessive on average so that selfing can also affect selection through a better purging of deleterious alleles in homozygotes. As a control, we run simulations with linked selection and varying degrees of selfing and applied polyDFE. In contrast with DFE-alpha (see Gilbert et al., 2022), we found that polyDFE tended to underestimate the proportion of weakly deleterious mutations (Supplementary Figure S11). We also found that the method did not estimate spurious signatures of beneficial mutations in outcrossers. Overall, the results were conservative to the effects of linked selection and selfing.

## Discussion

We compared the patterns of genetic diversity and selection across the genome in thirteen species with contrasted mating systems. We found far less polymorphism and far less selection efficacy in selfing than in outcrossing species, as observed in previous studies (e.g., Barrett et al., 2014; Burgarella et al., 2015; Chen et al., 2017; Glémin et al., 2006; Hazzouri et al., 2013; Laenen et al., 2018; Slotte et al., 2013). We also showed that these genomic effects depend on the interplay between linked selection and mating systems and vary with self-fertilization rates. For this, we leveraged a study design tailored to go beyond global patterns and decipher their underlying causes. First, differently from previous general comparisons among plant species (Chen et al., 2017; Glémin et al., 2006), we compared related species with similar LHTs, ecology, and genomic features, which allows more direct testing of the effect of the mating system. Second, we investigated all species of a clade covering a large range of mating systems, including intermediate mixed mating species, whereas previous studies addressing sister (or closely related) species mainly focused on extreme outcrossing vs selfing comparisons (e.g., Burgarella et al., 2015; Hazzouri et al., 2013; Slotte et al., 2013; Teterina et al., 2023). Third, by using recombination maps, we quantified the effect of linked selection in a comparative way.

Linked selection appears to be a main mechanism shaping levels of diversity in wild wheats, as it can reduce polymorphism to three to five-fold in outcrossing species (Figure 3B) and to one or two orders of magnitude in selfing species. This is in agreement with but higher than observed by Corbett-Detig et al. (2015), who found a quantitatively limited effect of linked selection except in selfing species. These quantitative values must be viewed with caution because it is difficult to properly fit a model of linked selection in selfing species. In addition to the complexity of the interaction between recombination, selfing and selection, it is not clear whether the species scale or a more local population scale is the most relevant. Another expected consequence of linkage and selfing is a reduction in selection efficacy, both against deleterious and in favor of beneficial mutations. We observed a striking contrast between regions of high and low recombination in SI species, with twice more weakly selected deleterious mutations and no beneficial mutation expected to fix in regions of low recombination (Figure 5; Supplementary Figure S10). In highly selfing species, instead, recombination had a weaker effect and only for deleterious mutations (Figure 5). Interestingly, the regions of high recombination in selfers exhibited a similar amount of weakly deleterious mutations as regions of low recombination in outcrossers, and we detected no signature of adaptive evolution at all in the two highly selfing species. It is worth noting that selfing violates the assumptions of the polyDFE underlying model and can distort the expected SFS through both the amplification of linked selection and the enhanced selection against homozygotes. However, simulations showed that these results were not artefactual and may even underestimate the effect of selfing (Supplementary Figure S11). Overall, the cross-comparison between mating systems and recombination levels clearly showed that the main quantitative effect of selfing is due to high linkage and linked selection.

Although our studied species share LHTs and have similar ecology, factors other than the mating system could affect genetic diversity, such as factors generating contrasted geographic ranges unrelated to mating systems. However, we showed that the geographic range has no effect on polymorphism patterns (Figure 2; Supplementary Figure S7). More generally, we cannot exclude that other factors could play a role, but they could hardly generate a very strong relationship between the mating system and the genetic diversity we observed. Strikingly, this strong relationship holds despite the use of an indirect measure of the selfing rates through phenotypic proxies.

Our results help better understand the evolution of selfing species. In the short term, selfing is known to recurrently evolve from outcrossing, depending on reproductive assurance and gene transmission advantage balanced by inbreeding depression, which can be partly purged during such transitions. In the long run, selfing lineages tend to diversify less than outcrossing ones, and selfing is considered an evolutionary dead-end, likely because of higher probability of extinction (Goldberg & Igić, 2012; Igic et al., 2008; Stebbins, 1957). The very causes of higher extinction rates in selfers remain unclear, but increased load and loss of genetic diversity and adaptive potential are possible drivers. However, the pace at which the effects of selfing manifest is still poorly known, although it is likely rapid as in the selfing *Capsella rubella* recently derived from the SI *C. grandiflora* (Slotte et al., 2013) or within the species *Arabis alpina* among populations with contrasted mating systems (Laenen et al., 2018).

In the *Aegilops*/*Triticum* genus, several instances of evolution towards different degrees of selfing likely occurred in a short evolutionary time period, which manifested by wide variations in floral traits associated with specific genetic diversity patterns. At the short phylogenetically scale we studied, we thus found a clear signature of the joint evolution of morphological traits and population genomic patterns, suggesting that the negative effects of selfing manifests rapidly. It is tempting to propose that the strong and rapid deleterious effects of selfing we detected will accelerate the extinction of the most selfing lineages. However, so far, there is no approach to properly test this hypothesis (Glémin et al., 2019a; Wright et al., 2013). Genomic degradation certainly accompanies the transition towards selfing, but we still do not know whether it is the ultimate cause of selfing lineages extinction.

Finally, our results also bear more general implications about the central question of the determinants of genetic diversity beyond the case of selfing species. In line with previous comparative analyses of polymorphism at the genome scale (Chen et al., 2017; Romiguier et al., 2014), we showed that LHTs, here the mating system, are a much stronger determinant of genetic diversity than proxies for census population sizes, here species range. Species range had no effect either after globally controlling for the mating system (Supplementary Figure S7) or after removing specifically the effect of linked selection (no significant correlation between species range and 𝜋_max_ or, if so, in the unpredicted direction). In contrast to previous studies, however, the range of genetic diversity is particularly large despite species being closely related and recently diverged (about 6 MYA), with similar genomes and life history traits (wind-pollinated annual herbs) except mating system. In these wild wheats, species nucleotide polymorphism varies with a factor of 20 (from 0.0011 to 0.022). For comparison, a clade of selfing and outcrossing *Caenorhabditis* nematodes species diverged less than 30 MYA show even wider disparities in polymorphism, up to a factor of 80 (Cutter, 2008; Li et al., 2014). In contrast, in a butterfly family that diverged around 120 MYA, with a four-fold variation in body mass and two-fold variation in chromosome numbers, only a factor of 10 was observed (from 0.0044 to 0.043) (Mackintosh et al., 2019). Similarly, among 28 species widely covering the seed-plant phylogeny and life forms (from annual herbs to trees), the observed range was only slightly higher than in wild wheats, with a factor of 28 (from 0.00064 to 0.018) (Chen et al., 2017). This points to mating systems as a main determinant of variation in genetic diversity among species.

However, variation in genetic diversity is still narrower than predicted from variation in species range, which varies by a factor of 500 here. This is in line with “Lewontin’s paradox,” the general observation that genetic diversity varies much less across species than census size does (Charlesworth & Jensen, 2022; Lewontin, 1974). Variation in 𝜋_max_ is around 60, so higher than variation in 𝜋_S_, around 20, but only by a factor three. Despite its strong effect, linked selection is thus unlikely to explain alone the limited range of variation in 𝜋_S_, in agreement with previous results (Buffalo, 2021; [Bibr CIT0010][Bibr CIT0017]).

## Supplementary material

Supplementary material is available online at *Evolution Letters*.

qrae039_suppl_Supplementary_Figures

qrae039_suppl_Supplementary_Tables

## Data Availability

Custom R and bash codes used for the analyses are available on https://github.com/sylvainglemin/ms-rec-triticeae along with input files. Software for genotype calling (reads2snps v. 2.0.64, ORF_extractor.pl) and polymorphism estimates (dNdSpNpS v.3) are available at https://kimura.univ-montp2.fr/PopPhyl/index.php?section=tools. Morphological trait measures are provided in Supplementary Tables S2 and S3. Filtered and cleaned sequence alignments to perform polymorphism analyses are available at https://bioweb.supagro.inra.fr/WheatRelativeHistory/index.php?menu=downloadMating. Raw data are deposited at the Sequence Read Archives (SRA) under project PRJNA945064 (submission number SUB12943046).
